# Magnetic resonance imaging, computed tomography, and ^68^Ga-DOTATOC positron emission tomography for imaging skull base meningiomas with infracranial extension treated with stereotactic radiotherapy - a case series

**DOI:** 10.1186/1746-160X-8-1

**Published:** 2012-01-04

**Authors:** Reinhold Graf, Michail Plotkin, Ingo G Steffen, Reinhard Wurm, Peter Wust, Winfried Brenner, Volker Budach, Harun Badakhshi

**Affiliations:** 1 Department of Radiation Oncology, Charité Universitätsmedizin Berlin, Berlin, Germany; 2 Department of Nuclear Medicine, Charité Universitätsmedizin Berlin, Berlin, Germany; 3 Department of Radiation Oncology, Klinikum Frankfurt (Oder), Germany

**Keywords:** Meningioma, Skull Base, ^68^Ga-DOTATOC, PET, Stereotactic radiotherapy

## Abstract

**Introduction:**

Magnetic resonance imaging (MRI) and computed tomography (CT) with ^68^Ga-DOTATOC positron emission tomography (^68^Ga-DOTATOC-PET) were compared retrospectively for their ability to delineate infracranial extension of skull base (SB) meningiomas treated with fractionated stereotactic radiotherapy.

**Methods:**

Fifty patients with 56 meningiomas of the SB underwent MRI, CT, and ^68^Ga-DOTATOC PET/CT prior to fractionated stereotactic radiotherapy. The study group consisted of 16 patients who had infracranial meningioma extension, visible on MRI ± CT (MRI/CT) *or *PET, and were evaluated further. The respective findings were reviewed independently, analyzed with respect to correlations, and compared with each other.

**Results:**

Within the study group, SB transgression was associated with bony changes visible by CT in 14 patients (81%). Tumorous changes of the foramen ovale and rotundum were evident in 13 and 8 cases, respectively, which were accompanied by skeletal muscular invasion in 8 lesions. We analysed six designated anatomical sites of the SB in each of the 16 patients. Of the 96 sites, 42 had infiltration that was delineable by MRI/CT and PET in 35 cases and by PET only in 7 cases. The mean infracranial volume that was delineable in PET was 10.1 ± 10.6 cm^3^, which was somewhat larger than the volume detectable in MRI/CT (8.4 ± 7.9 cm^3^).

**Conclusions:**

^68^Ga-DOTATOC-PET allows detection and assessment of the extent of infracranial meningioma invasion. This method seems to be useful for planning fractionated stereotactic radiation when used in addition to conventional imaging modalities that are often inconclusive in the SB region.

## Introduction

Meningiomas are common intracranial tumours with 25 to 30% located at the skull base (SB) [1]. When originating from the anterior clinoid process or medial sphenoid wing, they have an increased propensity to invade bone [2], which is a strong risk factor for recurrence [3]. Meningiomas in this location tend to progress transcranially or invade the infracranial spaces via natural openings [2,4] and recur in up to 45% of cases after surgery [5]. There is a strong correlation between the extent of resection and rate of recurrence [6]; therefore, accurate determination of tumour extension is critical for planning the magnitude of surgery and/or radiotherapy.

Computed tomography (CT) and magnetic resonance imaging (MRI) are widely used in the diagnosis of SB meningiomas and complement each other in the ability to determine tumour extent [7]. CT has proven to be more effective than MRI in diagnosing bone infiltrations at the anterior region of the SB [8,9]; while, MRI imaging of the cranial base has a high sensitivity due to excellent spatial and contrast resolution. However, especially in the central SB, there are structures with high signal intensity and high contrast-enhancement in MRI, which make it difficult to exactly delineate meningioma tissue from normal structures [10].

Receptor imaging offers an additional tool for imaging meningiomas. Meningiomas show high expression of several receptors, including somatostatin receptors (SR) subtype 2 (SSTR 2) [11,12]. Recently the somatostatin analogue, 1,4,7,10-tetraazacyclododecane-N,N',N",N"'-tetraacetic-acid-D-Phe1-Tyr3-octreotide (^68^Ga-DOTATOC) labeled with the positron emitter ^68^Ga (half-life, 68 min) was developed. ^68^Ga-DOTATOC is a positron emission tomography (PET) tracer and shows up to nine-fold higher affinity to SSTR 2, as compared to the SPECT ligand 111In-DTPA-octreotide [13]. In studies conducted by Henze et al., all meningiomas evaluated showed a high ^68^Ga-DOTATOC uptake [14,15]. In a pilot study, the same group recently performed CT, MRI, and ^68^Ga-DOTATOC PET examinations on 26 patients with intracranial meningiomas before radiotherapy. ^68^Ga-DOTATOC-PET provided additional information concerning the spread of the tumour and led to a significant modification of the gross tumour volume in about two thirds of the patients examined [16]. The acquisition of ^68^Ga-DOTATOC scans on PET/CT scanners helps to estimate the relation of PET findings to anatomical structures. In our own study using ^68^Ga-DOTATOC-PET/CT, the gross tumour volume was modified based on ^68^Ga-DOTATOC-PET data in 28/39 patients with positive PET scans [17].

In the present study, the results of MRI, CT, and ^68^Ga-DOTATOC PET were retrospectively compared in a study group of 16 patients with SB meningiomas. The influence of PET imaging on pretherapeutic detection of transgression of the SB was evaluated and the influence of PET on the definition of tumour extent was quantified.

## Materials and Methods

Between May 2006 and November 2010, a group of 50 consecutive patients with 56 SB meningiomas underwent planning CT, MRI, and ^68^Ga-DOTATOC-PET/CT (with contrast-enhanced CT) prior to the start of therapy. Fifty meningiomas showed areas with high ^68^Ga-DOTATOC uptake. Infracranial extension was visible in MRI/CT or PET in 16/50 patients, who formed the study group and were further analysed in a retrospective manner. There was infracranial extension in all patients. In addition, some patients showed extracranial extension to other sites (not infracranial, four orbit and one maxillary sinus). These extensions were not evaluated in this study. The study group included 11 women and 5 men with a mean age of 54.4 (range 25-73) years. Eleven patients had undergone surgery and/or radiotherapy and 5 patients had not received any therapy before. Pathohistologically, there were 7 meningiomas with a WHO grade 1 tumour (9 unknown). Nine meningiomas underwent FSRT as primary treatment without histological confirmation, when imaging morphology and clinical course suggested the diagnosis of a WHO grade I or II meningioma. The study was based on the Declaration of Helsinki and the principles of 'good clinical practice'. The protocol was approved by the ethics committee of our institution. Written informed consent was obtained from all patients before enrolment into the study. Written informed consent was obtained from the patient for publication of this case report and accompanying images. A copy of the written consent is available for review by the Editor-in-Chief of this journal.

Details of imaging the tumour volume for fractionated radiotherapy in the Charité medical school have been described elsewhere [17]. Briefly, following the planning-CT, MR imaging of the skull was performed with the use of a head coil in most patients with a 1.0 T scanner (Siemens Harmony™, Siemens Medical Solutions, Erlangen, Germany). Regularly, magnetization-prepared rapid gradient echo (MP-RAGE) T1-weighted sequences were used for coregistration after intravenous application of Gadolinium-DTPA ([Gd], Magnevist™, Schering AG, Berlin, Germany) at a dosage of 0.1 mmol/kg of body weight. These 3-D volume datasets at a 1- (to 1.5) mm slice thickness offer high spatial resolution and allow for coronal and sagittal reformations, enabling contouring in orthogonal planes.

Details of functional imaging have been described previously [17]. ^68^Ga-DOTATOC was applied intravenously followed by a tracer uptake phase of 60 min, as recommended by Henze et al. [15]. The applied dose of ^68^Ga-DOTATOC was between 70 and 120 MBq (1.9-3.2 mCi). The patient was placed in a dedicated positioning device for the head using an additional cushion and bandages for fixation. A contrast enhanced low-dose CT scan (detector collimation, 16 × 1.5 mm; tube current, 100 mAs; tube voltage, 120 kV; gantry rotation time, 0.8 s) of the entire head was performed for attenuation correction. PET was acquired in a single bed position with a 16 cm axial FOV from the base of the skull to the vertex and an emission time of 20 minutes. PET emission data were reconstructed as coronal, axial, and sagittal using a 128 × 128 matrix.

Planning-CT, MRI, and PET data were coregistered using the treatment planning software BrainSCAN™ v.5.1 (BrainLAB AG, Feldkirchen, Germany). CT, MRI, and PET were fused automatically using image fusion software and a mutual information algorithm. The validity of image fusion has been successfully tested previously by Grosu et al. [18].

The windowing of ^68^Ga-DOTATOC-PET was defined visually following the method published by Astner et al. [19]. The threshold of PET was adapted to tumours visible by MRI in regions where the tumour bordered normal brain tissue and could be outlined with high precision, e.g., where the meningioma bordered normal brain matter. Under the assumption that the ^68^Ga-DOTATOC-PET uptake in meningiomas is homogeneous [20], the tumour borders (defined on ^68^Ga-DOTATOC-PET images in slices with well defined borders by MRI) were used to outline the tumour margins on ^68^Ga DOTATOC-PET images in regions where the margins were not visible by MRI.

All examinations in one patient were performed within a time frame of 14 days. A checklist for local tumour extension and infiltration of bone and sites was used in this study. Conventional imaging findings were regularly interpreted by two experienced radiologists. Using a dedicated workstation, two experienced nuclear medicine physicians interpreted PET/CT fused images and their CT and PET components. If there was disagreement, the comparison results were reached by consensus.

The planning CT scans obtained with bone window settings (window width 2000 Hounsfield units, centre level 500 Hounsfield units) were used to determine the signs of erosion of adjacent bone or hyperostotic changes [21]. Tumour-specific abnormalities were defined as hyperintense or Gd enhancing structures in MRI and tracer enhancing areas in PET, often a "side-to-side" comparison with MRI was used to determine if a structure was normal or abnormal [22]. Visualization of bony structure changes, tumours spreading to the bony canals or foramina, communication with the middle fossa, and infracranial tumour expansion were compared in MRI, CT, and PET images. In an additional step, the infracranial volume was delineated using MRI/CT and ^68^Ga DOTATOC-PET separately. The statistical software R, version 2.11.1 (R Foundation for Statistical Computing, Vienna, Austria) was used for statistical analysis. Non-parametric differences were analysed using the Wilcoxon test (at a 0.05 level of significance).

## Results

All meningiomas showed areas with high ^68^Ga DOTATOC uptake, enabling delineation in MRI ± CT and PET in each case. The majority (75%) of lesions involved the sphenoid bone. Volumes measured by MRI were enlarged in MRI/CT by a mean additional volume of 2.3 ml (7.2% of the MRI volumes) that was only identifiable in CT. Osteolytic lesions represented the majority of bony lesions. Two meningiomas showed infracranial invasion along the vessels without bony abnormalities in CT. Evaluation of meningioma infiltration in the bony foramina by CT showed most tumorous changes were evident in the foramen rotundum and the foramen ovale (Table 1). In lesions accompanied by structural changes of these foramina, infiltrative growth into skeletal muscle was present in 8 lesions (62%).

**Table 1:** Patient characteristics and CT findings

**Pat. No**.	Gender	Age	Location	Bony changes (Location)	Bony changes (Type)	Bony changes (foramen ovale/rotundum)
1	F	47	L sphenopetral	SP/---/OC	Lytic	OV/RO

2	M	56	R petroclival	---/TE/---	Lytic	--/--

3	F	56	R sphenopetroclival	---/TE/---	Lytic	OV/RO

4	F	53	L spenoid ridge	SP/TE/---	Mixed	OV/RO

5	M	47	L sphenopetroorbital	SP/TE/---	Mixed	OV/RO

6	F	51	L sphenoidal	SP/TE/---	Lytic	OV/--

7	F	63	R sphenoidal	SP/TE/---	Hyperostotic	OV/RO

8	F	66	R sphenoidal	SP/TE/---	Mixed	OV/RO

9	M	63	L spheniodal	SP/---/---	Lytic	OV/--

10	F	57	L petroclival	SP/---/OC	Mixed	OV/---

11	M	25	L spheniodal	SP/TE/---	Lytic	OV/---

12	F	37	L temporobasal	---/---/---	NC	---/---

13	F	50	L sphenoorbtal	SP/---/---	Lytic	OV/RO

14	F	73	R sphenoorbital	SP/---/---	Hyperostotic	OV/RO

15	M	53	L petroclival	--/---/---	NC	---/---

16	F	74	R sphenopetroclival	--/---/---	Lytic	OV/---

F = Female; M = Male; L = Left; R = Right; SP = Sphenoid bone; TE = Temporal bone; OC = Occipital bone; NC = No changes; OV = Foramen ovale, RO = Foramen rotundum.

**Figure 1:**
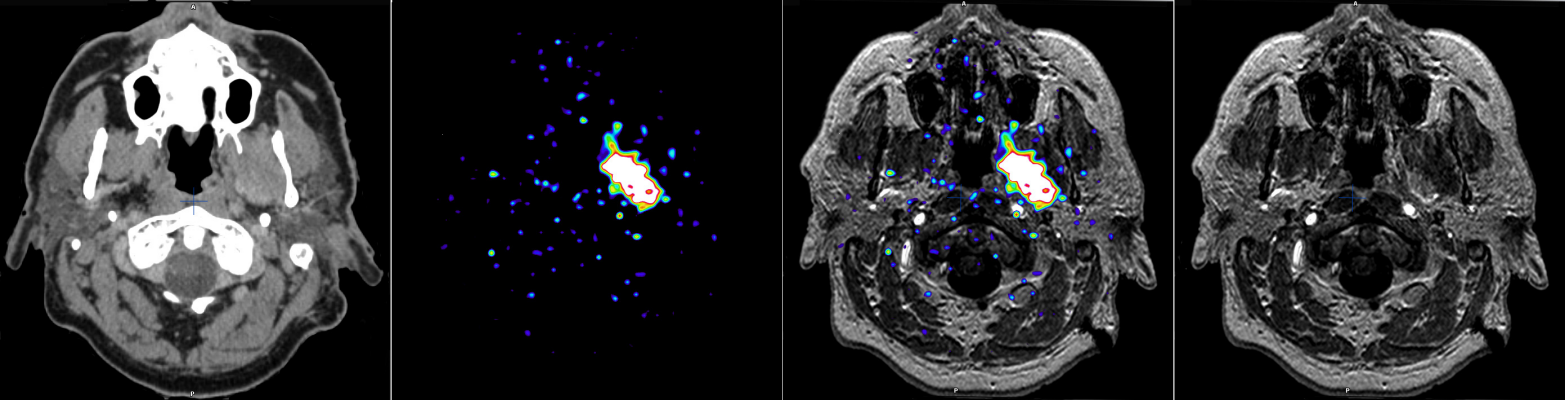
**A 49 year old male patient (No. 5) with a recurrent left petroclival meningioma with extension into the orbit, maxillary sinus, and intracerebral areas**. ^68^Ga-DOTATOC-PET/CT allowed markedly better delineation of the extent of infiltrative, infracranial extension compared with both CT and MRI.

The mean and median of the MRI/CT and PET volumes were almost identical. We analysed involvement of six designated infracranial spaces in each patient, resulting in a total of 96 sites. All 16 meningiomas grossly extended into at least one infracranial site. The infratemporal fossa and pterygopalatine fossa were mostly involved. The visualization of tumour expansion into designated infracranial spaces by MRI/CT and PET and a comparison between both modalities are shown in Table 2, with PET demonstrating a slightly better visibility of the involved areas compared with MRI/CT. With respect to the infracranial sites evaluated, there were 7 cases with negative MRI/CT and positive PET, resulting in discrepancies in 7 of the 96 sites evaluated (7%) and 7 of the 42 sites affected (17%). The infracranial volume delineable by PET was larger than the volume delineable by MRI, although it did not reach the level of significance (p = 0.06). Example images are shown in Figure 1.

**Table 2:** Comparison of MRI/CT and PET findings for detection of infracranial invasion in meningiomas with SB transgression on a lesional basis.

**Pat. No**.	Volume MRI/CT (cm^3^)	Volume PET (cm^3^)	Infracranial invasion	Infracranial invasion	Infracranial invasion	Infracranial invasion	Infracranial invasion	Infracranial invasion	Infracranial volume	Infracranial volume
			**ITF**	**PPF**	**Masticator space**	**Carotideal space**	**Para-pharyngeal space**	**Retro-pharyngeal space**	**MRI/CT (cm^3^)**	**PET (cm^3^)**

1	52.2	69.3				MRI/CT+PET	MRI/CT+PET	MRI/CT+PET	15.9	26.9

2	12.4	7.3				MRI/CT+PET	MRI/CT+PET		3.4	2.4

3	13.9	23.1	PET		PET	PET			0	0.3

4	21.7	10.6	PET	PET	PET				0.15	1.6

5	68.2	100.6	MRI/CT+PET	MRI/CT+PET	MRI/CT+PET		-		15.4	21.7

6	30.5	43.1	MRI/CT+PET	MRI/CT+PET	MRI/CT+PET				12.2	17.7

7	62.8	106.0	MRI/CT+PET	MRI/CT+PET	MRI/CT+PET				24.4	30.6

8	39.7	41.5	MRI/CT+PET		MRI/CT+PET		MRI/CT+PET		2.2	2.5

9	17.6	19.0	MRI/CT+PET	MRI/CT+PET					8.8	9.0

10	35.2	28.5	MRI/CT+PET			MRI/CT+PET			8.0	9.7

11	52.5	41.0	MRI/CT+PET	MRI/CT+PET	MRI/CT+PET				21.7	24.4

12	9.6	15.0	PET						0	0.3

13	4.6	3.5	MRI/CT+PET	MRI/CT+PET	MRI/CT+PET				10.0	6.0

14	76.0	67.8	MRI/CT+PET	MRI/CT+PET	MRI/CT+PET				9.3	6.1

15	27.1	8.0				MRI/CT+PET	MRI/CT+PET		0.3	0

16	19.6	39.4				MRI/CT+PET	MRI/CT+PET	PET	1.2	1.6

ITF = Infratemporal fossa; PPF = Pterygopalatine fossa.

## Discussion

Transcranial meningiomas spread through the foramina of the skull, entering the pterygoid region through the floor of the middle cranial fossa, suture lines, and the foramina of the skull [23,24]. This group of meningiomas is characterized by a high rate of recurrence, up to 45% in some studies [2,25,26]. Recurrence correlates with the extent of resection in neurosurgery [6] and extent of coverage in stereotactic radiosurgery [27].

Tumorous invasion of SB bone without hyperostosis was addressed in a study by Pieper et al. [28], where each of the eight patients showed erosion of the middle fossa floor and extension through the cranial base foramina, specifically the rotundum and ovale, without evidence of hyperostosis. The prevalence of lytic changes is in accordance with our findings. Of the 16 patients with infracranial extension, identified by MRI/CT or PET, there was association with bony changes in 13 patients (88%). These findings confirmed the observation of Leonetti et al. [2] that there was a strong correlation between radiologically visible invasion of osseous structures of the middle cranial and infracranial growth of meningiomas. Furthermore, in patients in whom structural bone changes were identified preoperatively, *histopathological *findings showed the tumour grossly invaded the skeletal muscle in all cases; while, this was only visible with *imaging *in 62% of cases in our study.

CT and MRI scanning have being estimated adequate for surgery for a long time [29,30]; although, anatomical imaging methods have their limitations for target delineation in infiltrative lesions and postoperatively [16]. We found the infracranial volume delineable by PET was larger than the volume by MRI/CT and the true extent of infracranial expansion could be missed by MRI/CT alone as 7/16 cases were positive by PET and negative by MRI/CT.

Other studies found ^68^Ga DOTATOC-PET improved the delineation of SB meningiomas compared with MRI/CT in the context of FSRT. Henze et al. [14] examined 8 meningiomas with ^68^Ga DOTATOC-PET and found valuable additional information regarding the extent of meningiomas located beneath osseous structures, especially at the SB. Later, the same group reported their experiences in 26 patients with meningiomas. They found 73% of the planned target volume for definitive radiotherapy treatment was significantly modified by the use of ^68^Ga-DOTATOC-PET [16]. In our previous study, which included 42 patients with meningiomas (27 SB), we used ^68^Ga-DOTATOC-PET/CT to detect modification of the gross tumour volume in 72% of patients [17]. The tumour extent visible by MRI/CT compared to ^68^Ga-DOTATOC-PET/CT was larger in 23% and smaller in 49% of cases. Dammers et al. described a case in which radioguided resection of a meningioma, using 111indium-labelled somatostatin receptors, enhanced the extent of the resection and discussed how this could be of potential use in maximizing the resection of meningiomas involving the cranial base region [31].

To our knowledge, descriptions of infracranial extension of SB meningiomas in the context of FRST are sparse. We reviewed the published studies concerning delineation of SB meningiomas with ^68^Ga-DOTATOC PET [14-17,32-34] and other tracers [19,35,36] and found transcranial extension of SB meningiomas was not mentioned, probably due to the comparably lower numbers of SB meningiomas examined.

When MRI/CT showed meningioma infiltration in the bony foramina, visualisation of the involvement by PET lacked small details, which may have been due to the higher slice thickness and lower spatial resolution of PET (in comparison to MRI/CT). Several other drawbacks in this study have been discussed previously [17]. As found in most studies on the target volume definition of meningiomas, this study is limited by the lack of histological verification. To address the problem of the choice of threshold levels [15,16], a fixed threshold could be useful for tumour segmentation to reduce interobserver variability. At our institution, re-evaluation of our data using an algorithm that creates defined SUV values is in progress.

## Conclusions

In this study, the extent of local meningioma invasion detected by MRI/CT and ^68^Ga-DOTATOC-PET were not consistent. ^68^Ga-DOTATOC-PET is a sensitive functional method for demonstrating the dimensions of infracranial meningioma infiltration and may contribute to FSRT planning in cases where CT and MRI are not conclusive in regions that are difficult to image.

## List of abbreviations used

MRI: Magnetic resonance imaging; CT: Computed tomography; SB: Skull base; MRI/CT: MRI ± CT; PET: Positron emission tomography; ^68^Ga-DOTATOC-PET: ^68^Ga-DOTATOC positron emission tomography; F: Female; M: Male; L: Left; R: Right; SP: Sphenoid bone; TE: Temporal bone; OC: Occipital bone; NC: No changes; OV: Foramen ovale, RO: Foramen rotundum; ITF: Infratemporal fossa; PPF: Pterygopalatine fossa.

## Competing interests

The authors declare that they have no competing interests.

## Authors' contributions

RG analyzed the CT, MRI, and PET data, performed the analysis, and drafted the first version of the manuscript. MP supervised the analysis of the PET data and revised the manuscript. RW made substantial contributions to the conception and design of the study. PW contributed to the analysis and interpretation of data. VB participated in designing the study and approved the treatment concepts. WB approved the final version of the manuscript. HB coordinated the recruitment of patients and data acquisition. All authors participated in critical discussion of the data and the conclusions. All authors improved the manuscript and approved the final version.
